# Expression of Angiogenesis Regulatory Proteins and Epithelial-Mesenchymal Transition Factors in Platelets of the Breast Cancer Patients

**DOI:** 10.1155/2014/878209

**Published:** 2014-10-14

**Authors:** Hui Han, Fang-Li Cao, Bao-Zhong Wang, Xue-Ru Mu, Guang-Yao Li, Xiu-Wen Wang

**Affiliations:** ^1^Department of Oncology, Qilu Hospital, School of Medicine, Shandong University, No. 107 Wenhua Xi Road, Jinan, Shandong 250012, China; ^2^Department of Oncology, Liaocheng People's Hospital and Liaocheng Clinical School of Taishan Medical University, Liaocheng 252000, China; ^3^Department of Oncology, Shandong Province-Owned Hospital, Shandong University, Jinan 250012, China; ^4^Department of Hematology, Liaocheng People's Hospital, Medical School of Liaocheng, Taishan Medical University, 67 Dong Chang Xi Lu, Liaocheng, Shandong 252000, China

## Abstract

Platelets play a role in tumor angiogenesis and growth and are the main transporters of several angiogenesis regulators. Here, we aimed to determine the levels of angiogenesis regulators and epithelial-mesenchymal transition factors sequestered by circulating platelets in breast cancer patients and age-matched healthy controls. Platelet pellets (PP) and platelet-poor plasma (PPP) were collected by routine protocols. Vascular endothelial growth factor (VEGF), platelet-derived growth factor BB (PDGF-BB), thrombospondin-1 (TSP-1), platelet factor 4 (PF4), and transforming growth factor-*β*1 (TGF-*β*1) were measured by enzyme-linked immunosorbent assay. Angiogenesis-associated expression of VEGF (2.1 pg/10^6^ platelets versus 0.9 pg/10^6^ platelets, *P* < 0.001), PF4 (21.2 ng/10^6^ platelets versus 10.2 ng/10^6^ platelets, *P* < 0.001), PDGF-BB (42.9 pg/10^6^ platelets versus 19.1 pg/10^6^ platelets, *P* < 0.001), and TGF-*β*1 (15.3 ng/10^6^ platelets versus 4.3 ng/10^6^ platelets, *P* < 0.001) differed in the PP samples of cancer and control subjects. In addition, protein concentrations were associated with clinical characteristics (*P* < 0.05). Circulating platelets in breast cancer sequester higher levels of PF4, VEGF, PDGF-BB, and TGF-*β*1, suggesting a possible target for early diagnosis. VEGF, PDGF, and TGF-*β*1 concentrations in platelets may be associated with prognosis.

## 1. Introduction

Breast cancer is a common malignancy in women and has a high mortality rate [1]. There are no predictive markers for metastasis or prognosis, although more attention has been paid to early detection of breast cancer metastasis.

The oncology community has long been interested in biological markers for tumor surveillance, early recurrence, and therapeutic efficacy. Tumor growth and metastasis depend on angiogenesis [2, 3] and angiogenesis regulators are considered ideal diagnostic markers and therapeutic targets. Studies have shown that monitoring levels of angiogenesis markers in biological fluids can be used to assess tumor growth and development [4–6] but they do not facilitate early diagnosis [7, 8]. There are studies showing that platelets play an important role in tumor angiogenesis and growth [9–14]. There are many regulatory proteins and proangiogenic endothelial cell growth factors in platelets, such as VEGF, PDGF, PF4, TSP-1, TGF-*β*, and bFGF [15–17]. Most of these proteins are released by platelets directly into the tumor microenvironment [18]. Studies of early phase for tumor formation in mice have shown increased levels of angiogenic proteins in platelets; however, there is no corresponding change in plasma [19]. It has been reported that VEGF was expressed in cancer patients' platelets [20]. However, it is unclear whether coexpression of multiple angiogenesis regulators in platelets is associated with breast cancer progress. In the present study, there is less study about relationship between these proteins and breast cancer prognosis.

In the present study, we aim to explore whether the increased protein levels in circulating platelets are associated with breast cancer progression and if these proteins can be biomarkers for breast cancer diagnosis. Therefore, we measured VEGF, TSP-1, PF4, and PDGF as well as analyzed the epithelial-mesenchymal transition factor TGF-*β*1 in breast cancer patients and healthy controls. In addition, we investigated the association between these indicators and clinical characteristics.

## 2. Materials and Methods

### 2.1. Study Population

Breast cancer samples at different tumor stages were collected from patients at the Surgery and Oncology Departments at Liaocheng People's Hospital (*n* = 37). Patients with platelet disorders or a medication history that would affect platelet physiology were excluded. Samples from the Department of General Surgery were collected before the operations. Samples from the Department of Oncology were collected at least 3 months after the conclusion of chemotherapy, radiotherapy, or medications for thrombocytopenia. The control group was selected from age-matched healthy female candidates with no history of platelet disorders or medications that may affect platelet function.

All samples were collected according to hospital guidelines and after obtaining informed consent in accordance with approved specimen collection protocols. Consent was given by 102 people for participation. The protocol was approved by the Ethics Committee of Liaocheng People's Hospital (number 2012126).

### 2.2. Clinical Characteristics

All patients were registered in the hospital admission system. Information regarding age, gender, surgical resection, tumor histology, hormonal receptor evaluation, and so forth could be accessed and collected from this system. Breast cancer staging was performed after surgery by histology and pathology according to the 2009 American Joint Committee Cancer Staging Manual. Hormonal receptors for estrogen (ER) and progesterone (PR) were regularly quantified by immunohistochemistry at the Department of Pathology, Liaocheng People's Hospital, following histology evaluation for breast cancer.

### 2.3. Sample Collection and Processing

Platelet isolation from whole blood was performed as described by Bergers et al. [21]. In brief, human venous blood (4 mL) was drawn into two tubes precoated with sodium citrate. Platelet-rich plasma was isolated by centrifugation of whole blood at 170 g for 15 min and then at 900 g for 10 min. PP were precipitated as white pellets and supernatants were retained as PPP samples. All samples were stored at −80°C.

Platelet pellet lysis was performed by using Abcam platelet isolation methods (isolation of human platelets from whole blood, Abcam). Lysis buffer (2x) consisted of 2% NP40, 30 mM Hepes, 150 mM NaCl, and 2 mM EDTA, at pH 7.4. Lysis buffer (50 *μ*L) was added to each pellet sample and vigorously mixed by pipetting and vortexing. PBS was used to dilute the lysed samples.

### 2.4. Measurement of VEGF, PDGF, TSP-1, PF4, TGF-*β*1, and Actin

Angiogenesis regulators were measured in PP and PPP. Patients with platelet disorders or a medication history that would affect platelet physiology were excluded from the study. All proteins were assessed by commercial ELISA kits according to manufacturer's protocols {human PDGF (HG04176), human TSP-1 (HG02106), human PF4 (HG021006), human actin (HG00954), human VEGF (HG06076), and human TGF-*β*1 (HG00468), IBL, Germany}. Preliminary experiments were performed to determine the optimal dilution range for each marker.

Activation of latent TGF-*β*1 was performed by acidifying with HCl and neutralizing with NaOH [22]. Briefly, each 100 *μ*L sample was added to precoated wells and incubated for 1-2 h at 37°C as described in the kit protocols. After washing, 100 *μ*L of conjugated secondary antibody was added and incubated for 1 h at 37°C or room temperature. The plates were washed three times; the substrate was added and incubated for 30 min at 37°C, followed by addition of the detection substrate and incubation for 25 min at room temperature. After a final wash, TMB substrate (100 *μ*L) was added, and the mixture was incubated at 37°C for 30 min. The colorimetric reaction was stopped after 30 min with 100 *μ*L stop solution. The results were analyzed by a Thermo Scientific microplate spectrophotometer at 450 nm.

### 2.5. Normalization of Protein Concentration and Quality Control

Actin levels were used to normalize the platelet contents in each sample. Protein contents in PP samples were also normalized to actin, thus eliminating potential bias introduced during processing and lysis. By associating platelet actin levels with platelet counts, we verified our method of PP lysis. A reference PP sample (platelet control), made from PPP samples, was used to monitor platelet lysis, dilution, and testing as a common reference for conversion from platelet counts to platelet protein level [23]. All standards and samples were run in duplicate and results were averaged.

### 2.6. Statistical Analysis

Median levels and interquartile ranges of each protein in breast cancer patients (*n* = 37) were compared to those of healthy controls (*n* = 65) using the nonparametric Mann-Whitney *U* test. Protein contents in PP were normalized and expressed per 10^6^ platelets; the results are shown in box-and-whisker plots [24]. Receiver operating characteristic (ROC) and area under the curve (AUC) with the Youden index were used to identify optimal cut-off values and evaluate the ability of each biomarker to differentiate cancer patients from controls [25]. Patients with platelet disorders or a medication history that would affect platelet physiology were excluded from the study. Pearson *χ* ^2^ test or Fisher's exact test was applied to identify differences in protein levels between patient groups. The Statistical Package for Social Sciences v. 16.0 was used for all statistical analyses and significance was defined as *P* < 0.05.

## 3. Results

### 3.1. Clinical Characteristics

The cancer patients represented four cancer stages and were aged 26 to 68 years. Platelet counts for all patients were within the normal range (105~295 × 10^9^/L). Clinical characteristics and ER/PR results are listed in Table 1.

### 3.2. Normalization to Platelet Count

**Figure 1:**
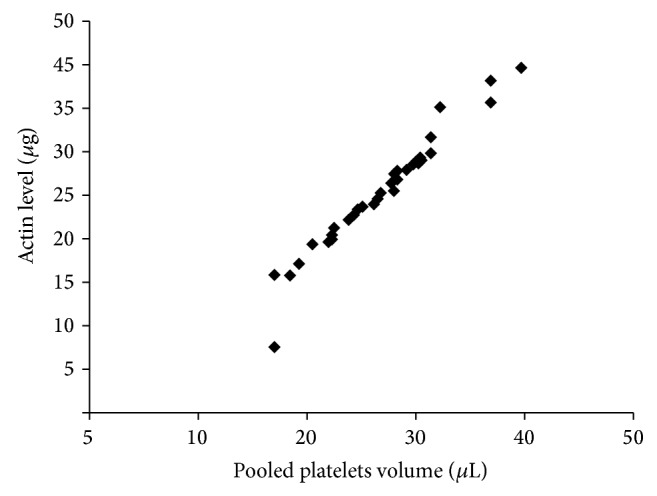
Dot plot of actin content and platelet count.

Actin concentrations were determined by ELISA and used to assess variations in PP volume after sample preparation. Actin quantities were similar between samples, demonstrating a close correlation between actin content and platelet count (Figure 1); however, we used the actin level in pooled platelets as a reference for protein normalization. Concentrations of all test proteins were calculated based on actin content and normalized to the actin level per 10^6^ platelets.

### 3.3. Platelet Sequestration of Angiogenesis Regulatory Proteins in Breast Cancer

**Figure 2:**
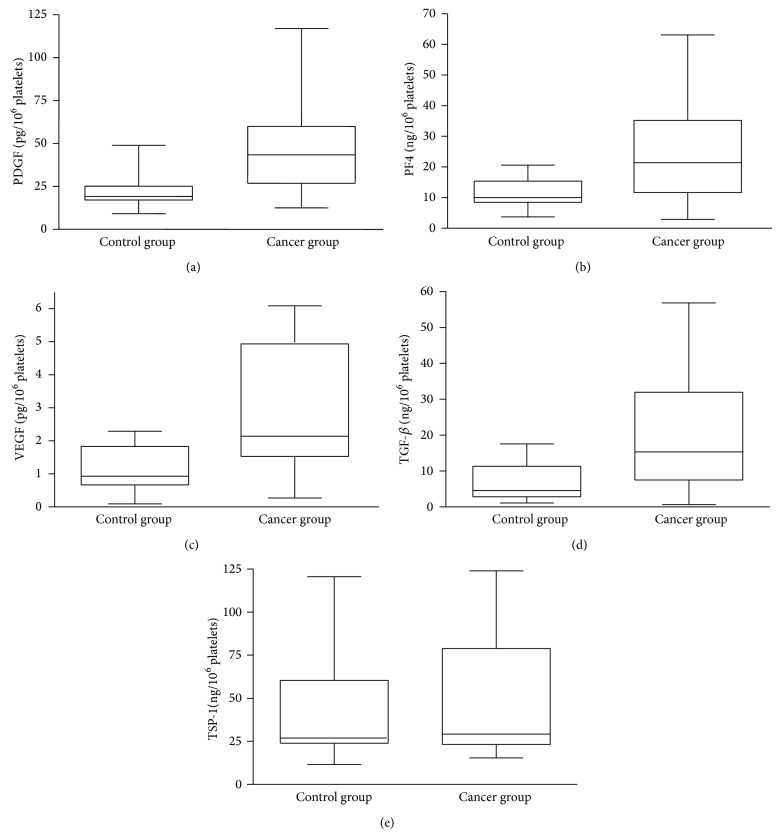
*Box plot of PDGF, PF4, VEGF, TSP-1, and TGF-*β* in PP*. The box indicates the lower and upper quartile and the central line is the median. The points at the ends of the whiskers are the 2.5 and 97.5% values. PDGF (a), PF4 (b), VEGF (c), and TGF-*β* (d).

PP and PPP from breast cancer patients and healthy controls were assessed. Compared with controls group, the median levels of VEGF, PF4, and PDGF-BB were significantly increased in PP from cancer patients (Table 2). Box-and-whisker plots for the cancer and control groups for four of the five biomarkers in PP illustrate elevated levels of PDGF-BB (Figure 2(a)), PF4 (Figure 2(b)), VEGF (Figure 2(c)), and TGF-*β* (Figure 2(d)). We also measured VEGF, PF4, PDGF-BB, and TGF-*β* in PPP samples. With the exception of TGF-*β*, there were no significant differences between the cancer and control groups (Table 3).

### 3.4. TGF-*β*1 in PP and PPP Samples from Cancer Patients

TGF-*β*1 is significantly elevated in cancer patients compared with healthy controls. Compared to the control group, the level of TGF-*β*1 in lysed PP was much higher (15.3 ng/10^6^ platelets versus 4.3 ng/10^6^ platelets, *P* < 0.001). However, in the PPP samples, TGF-*β*1 was lower in the cancer group than in the healthy controls; this maybe possibly due to sequestration of TGF-*β*1 by circulating platelets (24.2 versus 42.48 ng/mL, *P* < 0.001).

**Figure 3:**
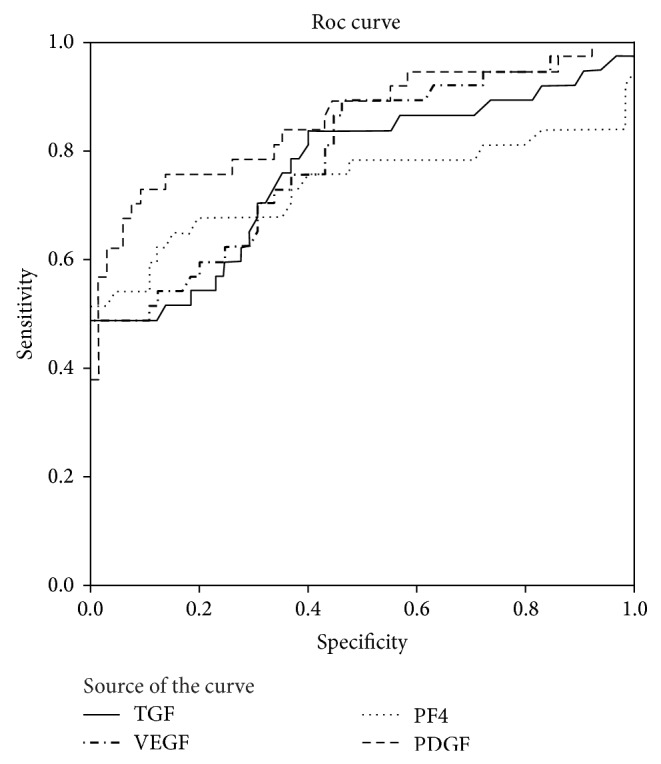
*Receiver operating characteristic (ROC) curves of PDGF, PF4, VEGF, and TGF-*β* in PP*. Dashed 45-degree line is the reference. ROC curves are based on continuous variables for each biomarker normalized to actin and expressed per 10^6^ platelets.

The level of PDGF (AUC: 0.856, 95% CI: 0.771–0.940), PF4 (AUC: 0.735, 95% CI: 0.612–0.859), VEGF (AUC: 0.789, 95% CI: 0.694–0.884), and TGF-*β*1 (AUC: 0.763, 95% CI: 0.658–0.868) in PP is significantly different between the cancer groups and control groups (Table 4). The ROC curve indicates the optimal cut-off points for each platelet biomarker (Table 5). Combined accuracy for all four biomarkers as a set yielded excellent discrimination between cancer and healthy controls in PP (*P* < 0.0001) (Figure 3).

### 3.5. Angiogenesis Protein and Epithelial-Mesenchymal Transition Factors Were Correlated with Clinical Characteristics

To investigate the correlation between clinical characteristics and protein accumulation in platelets, we divided the breast cancer patients into groups according to age (<40 versus 40–60 versus >60), cancer stage (stages I+II versus stages III+IV), hormonal receptor expression (low versus medium versus high). We found that the level of VEGF, PDGF-BB, and TGF-*β*1 in platelet was associated with clinical characteristics (Table 1).

## 4. Discussion

Numerous reports have suggested the activation role of platelets in cancer progression, tumor growth, and metastasis [26–28]. There are a large number of angiogenic proteins in platelets, including VEGF, PDGF, TGF-*β*1, angiopoietin-1 (Ang-1), and basic fibroblast growth factor (bFGF). These factors can promote vascular endothelial cell proliferation and formation of new blood vessels, which provide a suitable microenvironment for implantation and growth of tumor cells [29].

Recent study has found that the level of angiogenesis protein was increased in serum and tumor cells in patients with cancer [30–32]. Angiogenesis is a prognostic indicator for a variety of tumors [33]. However, the levels of many angiogenic proteins in plasma or serum can be detected until tumor load becomes large enough [34]. Angiogenesis markers quantification in breast cancer and their correlation with many clinicopathological prognostic variable angiogenic proteins are present in much higher concentrations in the platelets of experimental animals with malignant tumors than in the plasma, serum, and nonmalignant tumors [19]. Thus, regulation protein in platelets is more sensitive than that of them in plasma [35].

Our current data indicated that VEGF, PF4, PDGF-BB, and TGF-*β*1 levels were elevated in PP of the cancer group but not in PPP. These were consistent with the previous study of McDowell et al. [36], and we expanded the number of breast cancer patients on the basis. For detection of these proteins, we found that the curve of ROC was more than 0.7, indicating that these proteins exhibit high sensitivity and specificity. They seem to be potential candidates for breast cancer diagnosis.

Zaslavsky et al. demonstrated that the expression of TSP-1 in platelet is increased in a mouse model with Lewis lung cancer cell lines [37], suggesting that TSP-1 is an inhibitor of tumor angiogenesis in the early stages of tumor growth. However, we found that there is no significantly different of TSP1 expression between the cancer groups and control groups. The reason for this phenomenon maybe is the difference in tumor burden between animals and human patients. However, the sample size is small and the results must be interpreted with caution.

In the present study, TGF-*β*1 sequestration was increased in PP but decreased in PPP, although changes of concentration in PP are more robust. This may be due to elevated sequestration of TGF-*β*1 by platelets in cancer patients.

Angiogenesis factors are strongly correlated with the prognosis and clinical pathology, especially for breast cancer [38–40]. VEGF expression is higher in breast cancer tissue and is closely associated with ER labeling index, lymph node metastasis, clinical stage, and histological grade [41]. We are also trying to understand whether the concentrations of vascular regulatory proteins in platelets are also linked to cancer status. All four stages of breast cancer samples were studied. We found that the level of VEGF, PDGF, and TGF-*β*1 was associated with lymph node metastasis, clinical stage, and molecular subtype. Molecular portraits of human breast tumors are usually divided into four subtypes: luminal A, luminal B, HER-2(+), and basal-like [42]. Luminal A is usually associated with better prognosis, but HER-2(+) and basal-like subtypes indicate poor prognosis [43]. The result reported here reveals that the level of VEGF, PDGF, and TGF-*β*1 was higher than the latter two subtypes. This result is consistent with previous findings that ER-, PR-negative breast cancer has a high microvascular transfer rate and blood transfer risk [44, 45]. The correlation between these markers and the clinical characteristics of cancer may play an important role in breast cancer diagnosis and prognosis. Compared with tissue immunohistochemistry, ELISA method is simpler and less invasive than immunohistochemistry and is easy to perform.

In conclusion, we found that circulating platelets in breast cancer sequester higher levels of PF4, VEGF, PDGF-BB, and TGF-*β*1, and these proteins can be possible indicators for breast cancer in early diagnosis. The level of VEGF, PDGF, and TGF-*β*1 expression may be associated with cancer prognosis. Further analysis of protein expression in platelets before and after cancer treatment (surgery, chemotherapy, or radiotherapy) is needed to evaluate their utility as markers for disease monitoring.

## Figures and Tables

**Table 1:** Biomarker levels in PP and clinical characteristics.

Groups	Number of patients	PF4 (ng)	TGF-*β* (ng)	VEGF (pg)	PDGF-BB (pg)
Stage					
I+II	24	21.6 ± 11.3	16.5 ± 10.6^a^	2.37 ± 1.0^a^	40.4 ± 13.3^a^
III+IV	13	24.4 ± 11.5	24.6 ± 12.2	3.38 ± 1.28	49.9 ± 13.8
Age					
>60	8	22.9 ± 11.8	23.1 ± 12.5	2.84 ± 1.12	50.3 ± 14.6
40–60	23	22.3 ± 11.6	20.16 ± 17.4	3.05 ± 1.23	46.3 ± 13.5
<40	6	23.3 ± 12.9	20.8 ± 18.0	2.89 ± 1.11	44.6 ± 13.1
LN status					
N0	11	22.1 ± 11.2	16.4 ± 10.1^b^	2.32 ± 1.05^b^	40.2 ± 13.1^b^
N13	26	22.8 ± 12.1	24.5 ± 11.3	3.31 ± 1.14	49.8 ± 13.5
Molecular subtypes					
Luminal A	9	21.9 ± 11.7	18.4 ± 11.6^c^	2.66 ± 1.09^c^	42.8 ± 13.4^c^
Luminal B	12	22.1 ± 12.3	19.7 ± 12.9	2.73 ± 1.17	43.2 ± 13.3
HER-2(+)	7	24.9 ± 12.9	28.9 ± 13.7	3.51 ± 1.25	52.1 ± 14.3
Basal like	9	22.2 ± 11.1	33.2 ± 15.2	3.74 ± 1.26	56.2 ± 14.7

Variables are normalized to actin and expressed per 10^6^ platelets. Being constrained by relatively low number of candidates in each clinical subgroup, we used average content instead of median level to make the comparisons. ^a^Stages I+II versus III+IV: *P* < 0.05, ^b^LN status N0 versus N1-3: *P* < 0.05, and ^c^Luminal A or B versus HER-2 or Basal like: *P* < 0.05.

**Table 2:** Levels of biomarkers in PP of the cancer and control study groups.

Variable	Cancer group		Control group		*P* value
	(*n* = 37)		(*n* = 65)		
	Median	Range	Median	Range	
PDGF, pg	42.9	13.5–116.4	19.1	9.3–48.9	<0.001^*^
PF4, ng	21.2	3.1–63.2	10.2	4.2–20.5	<0.001^*^
TSP-1, ng	29.2	15.4–123.9	27.0	11.5–120.5	0.821
TGF-*β*, ng	15.3	0.4–56.0	4.3	0.5–16.9	<0.001^*^
VEGF, pg	2.1	0.3–6.1	0.9	0.1–2.3	<0.001^*^

Variables are normalized to actin and expressed per 10^6^ platelets.

^*^Statistically significant.

**Table 3:** Biomarker levels in PPP.

Biomarker	Units	Cancer group		Control group		*P* value
		(*n* = 37)		(*n* = 65)	
		Mean	SD	Mean	SD
PDGF	pg/mL	236.9	79.6	263.2	63.1	0.076
PF4	ng/mL	269.3	83.8	302.4	85.0	0.077
TSP-1	ng/mL	418.8	101.6	395.9	102.6	0.448
VEGF	pg/mL	53.8	16.1	53.3	11.1	0.669
TGF-*β*	ng/mL	24.2	5.9	42.48	10.0	<0.001^*^

Variables are not normalized.

^*^Statistically significant.

**Table 4:** ROC curves of biomarkers of PP in the cancer group.

Biomarker	AUC	Standard error	*P* value	95% CI	
				Lower limit	Upper limit
PDGF	0.856	0.043	0.001	0.771	0.940
TGF-*β*	0.763	0.054	0.001	0.658	0.868
VEGF	0.789	0.048	0.001	0.694	0.884
PF4	0.735	0.063	0.001	0.612	0.859

All *P* values were less than 0.05.

**Table 5:** Optimal cut-off point of biomarkers of PP in the cancer group.

Biomarker	AUC	Optimal cut-off	Sensitivity (%)	Specificity (%)
PDGF pg	0.856	26.65	75.7	86.2
TGF-*β* ng	0.763	7.55	70.5	69.2
VEGF pg	0.789	1.655	70.3	69.2
PF4 ng	0.735	15.3	67.6	80.0

Variables are normalized to actin and expressed per 10^6^ platelets.
